# Nahua biocultural richness: an ethnoherpetological perspective

**DOI:** 10.1186/s13002-021-00460-1

**Published:** 2021-05-12

**Authors:** Miriam Itzel Linares-Rosas, Benigno Gómez, Elda Miriam Aldasoro-Maya, Alejandro Casas

**Affiliations:** 1 El Colegio de la Frontera Sur, ECOSUR, Carretera Panamericana y Periférico Sur s/n Barrio María Auxiliadora, 29290 San Cristóbal de Las Casas, Chiapas Mexico; 2 Instituto de Investigaciones en Ecosistemas y Sustentabilidad, IIES, Universidad Nacional Autónoma de México, Antigua Carretera a Pátzcuaro 8701, 58190 Morelia, Michoacán Mexico; 3 CONACYT-El Colegio de la Frontera Sur, ECOSUR, Carretera Panamericana y Periférico Sur s/n Barrio María Auxiliadora, 29290 San Cristóbal de Las Casas, Chiapas Mexico

**Keywords:** Nahua contemporary knowledge, Herpetofauna, Sierra Negra, Tehuacan-Cuicatlán Valley, Ethnozoology

## Abstract

**Background:**

Mexico harbours one of the greatest biocultural diversities of the world, where multiple social and natural elements and systems form complex networks of interactions in which both culture and nature are mutually influenced. Biocultural states and processes are studied by ethnosciences, among them ethnoherpetology, which seeks understanding material and non-material expressions of the interactions between humans, amphibians, and reptiles. Herpetofauna has been part of the magic–religious world and source of goods for Mesoamerican cultures. This study aims to document and analyse the complex body of knowledge, beliefs, and practices on these vertebrates in the Nahua culture, the factors that have influenced progressive risk and loss of culture, habitat, and species, and the potential contribution of contemporary Nahua knowledge to biocultural conservation.

**Methods:**

Through 15 workshops with children and young people, and 16 semi-structured interviews to people 27 to 74 years old, we documented the contemporary Nahua knowledge in the communities of Aticpac and Xaltepec in the Sierra Negra, Puebla, central Mexico. Biological and ecological knowledge, use, management practices, legends, and perceptions on herpetofauna were emphasised in the study.

**Results:**

We obtained an ethnoherpetological checklist, grouping species into four general classificatory categories: *kohuatl* (serpents), *kalatl* (frogs and toads), *ayotsi* (turtles), and *ketzo* (lizards and salamanders), which included 21, 10, 1, and 11 ethnocategories respectively, based on the local Nahua knowledge of herpetofauna. Serpents, used as medicine, are the most culturally relevant. Due to perceptions of danger, beliefs, and actual snake bites, the main interaction with serpents is their elimination; however, some snakes are tolerated and maintained in captivity. The remaining species of local herpetofauna recorded are tolerated. Cultural aspects of reptiles and amphibians in the Nahua worldview were documented to influence the regulation of interactions of people with these vertebrates, but for younger generations, such aspects are less frequent or absent.

**Conclusions:**

Interactions and cultural relationships between the Nahua people, amphibians and reptiles are complex, maintaining some aspects of the local worldview but also influenced by external factors and being constantly recreated and re-signified. Documenting and understanding the contemporary relations is essential to generate strategies in biocultural conservation of herpetofauna.

**Supplementary Information:**

The online version contains supplementary material available at 10.1186/s13002-021-00460-1.

## Background

### Biocultural diversity and contemporary indigenous knowledge

The paradigm of biocultural diversity proposes that the biological and cultural diversity geographically coexisting, determine deep complex interactions that make nature and human societies interdependent; these interactions are especially marked among rural people and local or indigenous groups, which strongly depend on nature for their subsistence [1, 2]. By the end of the XIX Century, a considerable number of scholars and scientific approaches realized about the close relationships existing between human societies and their surrounding natural environment [2]. But more recently, the recognition and relevance of the study of such interactions and relationships has gained great interest [2, 3]. Interactions between humans and ecosystems may generate genetic and morpho-physiological diversity in managed organisms, ecosystems, and landscapes that are moulded to human needs through domestication [4]. In addition, they have generated a great cultural diversity in aspects of the daily life like food, health, art, production systems, beliefs, customs, festivities, housing, clothing, and religious celebrations, among others [1, 5]. Several authors have proposed that the biocultural complex is a crucial factor of resilience before natural and social problems, and for stopping the current socio-environmental crisis represented in the loss of species and habitats, ecosystem degradation, global warming, climate change, inequality, poverty, cultural erosion, and global health crisis, among others. It is therefore crucial to know, strengthen, and recover both biological and cultural diversity, since nowadays it is impossible to conserve one without the other [2–4, 6–8].

Throughout their history, human communities have constructed complex systems of knowledge (*corpus*), practices (*praxis*), and beliefs (*kosmos*), coupled to specific natural contexts that have allowed them to satisfy their material and spiritual needs [9] and to react before dangers, risks, and disasters. Adaptive processes of such knowledge and its cultural transmission have been determinant for peoples’ [10]. Berkes [10] has called this complex system the Traditional Ecological Knowledge (TEK), ahead we will use the term local ecological knowledge (LEK) [11]. On this basis, Toledo and Barrera-Bassols [7] have constructed their theory of ethnoecology, but the premises are also helpful for ethnosciences and general studies of culture [12]. LEK expresses not only what people know, but also their representations; non-material expressions that generate collective identity, norms of behaviour towards natural environment, values, forms of use and management of natural resources, and alternatives before stressful natural events [3, 7]. Based on Santos [13], Aldasoro-Maya [14] proposed adding the term “contemporary knowledge”, considering the importance to recognize that in human communities, different knowledge and experiences commonly coexist, some of which (generally those indigenous and local) are considered to be elements from the past [15, 16]. Therefore, the term “contemporary” emphasizes that, although the local/indigenous knowledge has its basis on traditional knowledge generated through processes occurring in thousands of years, it has been and currently is dynamic, under continuous production and reproduction of elements, open, adopting, and adapting before changing factors and different forms of knowledge it coexists with [14, 15, 17–19]. Another element of knowledge is environmental perception which is also dynamic, influenced by gender, generations, culture, and social and environmental history [20]. Environmental perception is formed by ideas, judgement, and values constructed on evaluations charged with affective perspectives, positive or negative attitudes towards environmental aspects and relations between humans and ecosystems [21]. The analysis of environmental perceptions reveals subjective aspects, opinions, beliefs, and norms that people establish in their relation with the natural environment [21]. Contemporary indigenous/local knowledge includes key elements to reinforce the sense of common property which is fundamental to generate appropriate proposals for biocultural conservation [14]. This knowledge includes a great diversity of information and experience on using a wide variety of organisms (plants, animals, and fungi, among others) [5, 7], ecosystems and landscapes, and a different worldview, conceptualizing them and relating to nature.

### The Tehuacán-Cuicatlán Valley, its biocultural diversity and ethnobiology

The Tehuacán-Cuicatlán Valley (TCV) and surrounding areas are a region of great biocultural diversity. Physiography, geology, and vegetation conform an extraordinary heterogeneity of ecosystems harbouring a large number of species and endemism [22]. It has also been the setting of a long cultural history, commonly referred to as one of the most ancient areas of agriculture and domestication of the Americas [12]. Currently, the cultural diversity of this region is represented by eight native ethnic groups (Nahua, Popoloca, Mixtec, Ixcatec, Chocholtec, Cuicatec, Mazatec, and Chinantec) [12]. Studies on floristic richness, plant use, and management have been prolific, but the faunistic studies have been limited. However, the information available reveals the existence of a high animal species richness. Although ethnozoological studies are still scarce, ethnobiological studies in the Sierra Negra, in the state of Puebla, inhabited by Nahua people are in progress [23, 24], and this study forms part of this effort.

### Ethnoherpetology: herpetofauna and Mexican cultures

Ethnoherpetology, directed to document, analyse, and understand the relation between humans, amphibians, and reptiles [25, 26], is probably one of the ethnozoological approaches less studied in Mexico, where biological diversity of these vertebrates is high. It has been estimated that in Mexico, there are 864 species of reptiles (417 lizards, 393 serpents, 48 turtles, three amphisbaenides, and three crocodiles), representing 8.7% of the reptile species of the world. In total, 376 species of amphibians have been recorded (234 frogs and toads, 137 salamanders, axolotls and two caecilians) [27, 28]. Puebla is one of the states of Mexico where the records of herpetofauna are among the highest, with 246 species representing 22.7% of amphibians and reptiles of this country, 11 species being endemic [29]. However, García-Vásquez et al. [29] state that 72% of species in Puebla (176 of them) have problems for conservation due to habitat loss and contamination, among other factors. In the Sierra Madre del Sur, where we conducted our study, herpetofauna is extraordinarily rich and significantly contributes to the species richness recorded for the TCV. In this area, 48 species of amphibians and 113 of reptiles have been reported. The Mexican Official Norm (NOM-059-2010) indicates that four species of amphibians and 16 of reptiles are threatened, while eight and 42 respectively are under special protection [29].

The herpetofauna diversity of Mexico explains that since pre-Columbian times, amphibians and reptiles were important components of the Mesoamerican cultures [25, 26, 30–32]. In their worldviews, serpents, crocodiles, turtles, lizards, frogs, toads, axolotls, and salamanders are outstanding. Even after centuries of domination and changes of cultures of the region, herpetofauna is still important [30, 33–35], being part of myths, legends, magic–religious beliefs, and symbolism, as well as food, medicine, clothing, handcrafts, ornaments, amulets, and even pets [35].

Ávila-Nájera et al. [30] reviewed uses and values of herpetofauna in México between 1997–2017, reporting that nearly 11% (103 of 864 species) of reptile species and 8% (32 of 376 spp.) of amphibians are used, mainly as medicine and food [30].

Among the Nahua, serpents are associated to important deities like Quetzalcoatl (the feathered serpent) and Cipactli, a crocodile deity associated to “Mother Earth” [32]. By the eighteenth century, it was recorded that the Nahua had a wide biological knowledge of some reptiles, outstandingly lizards [36], which were part of indigenous medicine, used to cure bites of poisonous animals and blindness, among other health problems (García de la Vega, cited in [36]). The importance of these animals is currently active; recently, it was documented that the Nahua from the Sierra Norte de Puebla classified serpents in the *kouamej* family that includes 17 ethnogenera [37]. According to people of that region, serpents take care of the milpas and water sources, warning and punishing people with bad behaviour [37]. The Nahua of Morelos consider *Phrynosoma taurus* to give good luck and abilities to people, and when put on the hand of a girl, it confers her the ability to make good maize tortillas [38]. The black iguana and rattlesnakes provide medicine and food, the mazacuata (*Boa constrictor*) is ornamental, the shell of the turtle *Kinosternon integrum* is ornamental and amulet for good luck and wards envies off. The frogs *Agalychnis dacnicolor* and *Lithobates spectabilis* are edible and pets [38].

In the TCV and surrounding areas, some uses and forms of management of herpetofauna have been documented among the Nahua [23]; however, a deeper documentation of ethnozoology of the region in general and ethnoherpetology in particular is still needed. Therefore, this study aimed to document and understand beliefs, knowledge, perceptions, and practices on herpetofauna by the Nahua people of the communities of Aticpac and Xaltepec and their potential contribution to biocultural conservation of the area.

## Methods

### Study area

**Fig. 1:**
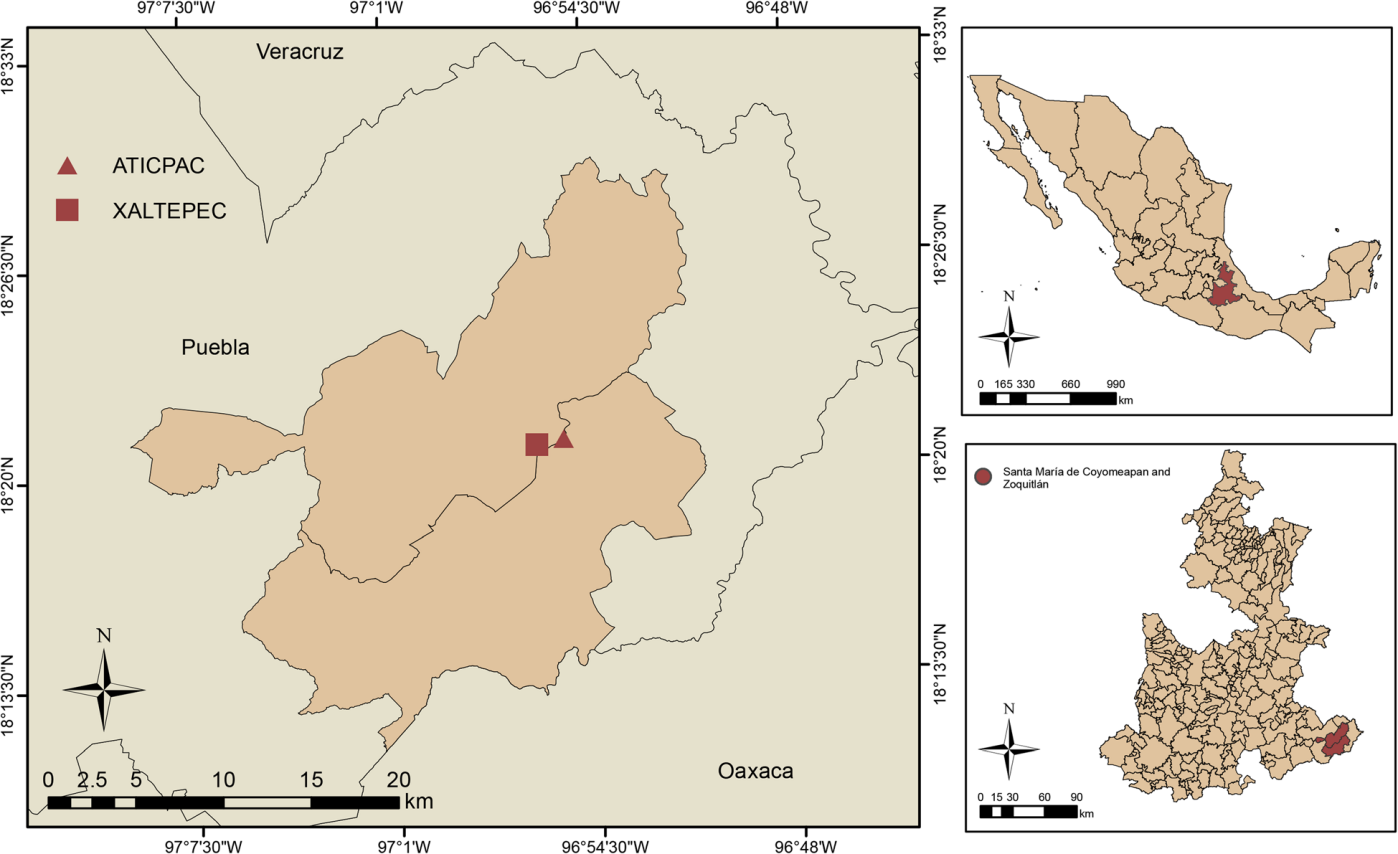
Location of Aticpac and Xaltepec, in the Sierra Negra of Puebla

Aticpac and Xaltepec belong to the municipalities of Santa María Coyomeapan and Zoquitlán, Puebla, respectively. Coyomeapan has a territory of 229 km^2^ [39], founded by the Popoloca people, while Zoquitlán has a territory of 268.87 km^2^, founded by the Nonoalca-Chichimeca and the Mazatec people, but conquered by the Nahua from Zoquitécatl in 1536 [39]. Both communities are part of the Sierras Orientales, in the province of Sierra Madre del Sur [40], belonging to the Region VII that includes the Tehuacán Valley and the Sierra Negra [39] (Fig. 1). Settled on sedimentary rocks, the predominant soil is luvisol [40]. Climate is semi-warm wet, with rains throughout the whole year [40, 41], annual temperatures average 20–22 °C, and annual rainfall 3000 to 3500 mm [40]. Vegetation is tropical rain forest. People living in the area are Nahua [42], who speak the dialectal variant of the Sierra Negra [43]. In 2010, Aticpac had a population of 152 persons (79 women and 73 men) [42], while Xaltepec had 155 persons (76 women and 79 men) [42]. Young people migrate to the cities of Tehuacán, Puebla, and México City. The main economic activities are agriculture, coffee cultivation, commercialization of non-timber forest products (mainly inflorescences of the tepejilote palms, *Chamaedorea tepejilote*, and tropical fruits) and fruit of cultivated trees. Some households raise goats, and others are carpenters. There is one kindergarten, two primaries, and one secondary schools.

### Field study methods

For documenting Nahua knowledge of herpetofauna, we carried out workshops with children and young people, as well as semi-structured interviews to young and adult people, since knowledge and perceptions of relations between humans and herpetofauna, mainly reptiles, may vary. We generated a respectful approach based on what Larrosa [44] called epistemic displacement of intervention to treat, in which symmetry is generated through horizontality experience based on developing attitudes of empathic interaction with the others. Such an approach provides key elements to ease documenting knowledge, beliefs, and practices from different intellectual and cultural platforms [45], and detailed understanding of the perspectives and experiences that people studied live [46]. It was our intention to carry out participatory sampling and dialogues for biocultural conservation, but because of the pandemic of COVID-19, we interrupted our fieldwork.

### Workshops

In the “Cuauhtémoc” bilingual primary school of Aticpac, we conducted nine meetings 2 h long each. It was a multilevel group formed by 15 boys and 7 girls between 6 and 12 years old. To identify the species of higher cultural relevance, we carried out a group free list, stimulating the interaction through images of local animals to increase the list of species. Through ludic activities (playing games like “duck, duck goose”, “split cheese”, and “roosters and hens”, which consisted of making circles, forming teams, making a chant, running, and winning another person’s place or catching a friend; then, a question or comment was asked to the person who lost the game to share something related to an ethnoherpetological topic and artistic labours (group discussions, participatory mapping, drawing, and storytelling); we investigated ecological, biological, and utilitarian knowledge, as well as management practices on these animals. Documenting myths and legends was stimulated by sharing Nahua legends (e.g., those of Quetzalcoatl and Axolotl, and a local legend on Quetzalcoatl previously recorded) which allowed a dialogue with children.

Activities with young people were carried out in the indigenous secondary school from Xaltepec, with the participation of 14 girls and 9 boys between 12 to 15 years old. We conducted six meetings 1 h long each. For documenting nomenclature and biological and ecological knowledge, we projected images of local herpetofauna and asked to write for each animal recognized its name in Spanish and Náhuatl, the site where they have seen it, the season, and all aspects and facts they remembered about each animal. For documenting myths and legends, we followed a similar method with the children.

### Interviews

We carried out 16 interviews of 40 to 90 min long. Most of them (12) were conducted with men from 27 to 74 years old, dedicated to agriculture, commerce, carpentry, and subsistence hunting. One of the interviewees is an expert in hunting and serpent remedies topics. Participation of women was limited since most of them said not to know about these animals, or they preferred their husband to talk about it. However, some visits to women were arranged, but only two could be made with women 40 years old (one housewife and the other a merchant). Regarding young people, due to the time constraints caused by the COVID-19 pandemic, only two interviews were conducted with 13-year-old boys from Xaltepec.

### Species identification

Although we could not carry out participatory sampling, we could identify serpents from samples maintained in alcohol for remedies, which were facilitated by six persons from Aticpac and Xaltepec. We had access to 18 specimens, and identified 10 species based on the guides published by [47–50]. In addition, we examined five specimens of turtles used for magic–religious purposes, all specimens corresponded to one single species.

### Quantitative and qualitative analyses

Data of the free lists generated with young and adult people were captured in Excel and analysed with the FLARES (Free List Analysis under R Environment using Shiny) programme. We calculated the relative cultural salience index (relative frequency of mention) as well as the Sutrop index or index of cultural relevance (combining both frequency and order of mention). This index uses the formula *S* = *F*/*N*mp, where *F* is the frequency of mention of each ethnocategory, *N* is the number of people interviewed, and mp is the medium rank of cites of each ethnocategory [51]. Values of this index goes from 0 to 1, with values closer to 1 indicating that ethnocategories have higher cultural relevance.

Since the primary school group is formed by children of different scholar degrees (including children that start learning to write), it was not possible for us to obtain personal listings, so we obtained a group listing that cannot be analysed through the Sutrop index.

Through the Atlas.ti 8. software for qualitative analysis, we coded and categorized the interviews. With those codes, we constructed a semantic net in which we analysed the complexity of the material and non-material relations that the Nahua have generated with the herpetofauna of the zone.

## Results

Nahua knowledge, practices, and beliefs on amphibians and reptiles in the communities of Aticpac and Xaltepec are varied and dynamic. We documented biological, morphological, and ecological knowledge, as well as different aspects on their attributes, properties, functions, and other aspects related to their use, management, perceptions, attitudes, and beliefs, resulting in complex relations over material and non-material aspects of people from local and neighbouring areas with herpetofauna. People know habitats, distribution ranges, reproduction seasons, activity schedules, and food types, among other aspects of the recognized species (see the next quote):

“There are not *mazacohuatl* in here, but there are in Temazcalco. It eats gophers, where it is present, there are no gophers” (Serafín 63 years old)*.*

Based on morphological features (colours, general aspect, size, and form of some structures, among others), ethological (sounds, behaviour, habits) and ecological aspects (distribution, habitat types, reproduction seasons, type of food), a total of 43 ethnocategories and subcategories were identified, which include 106 species (25 families belonging to four orders), some of them occurring in the zone (Supplementary material 1).

Four main classificatory categories were identified: *kohuatl* (serpents), including 21 ethnocategories representing 50 species; *ketzo* (lizards and salamanders), including 11 ethnocategories representing 25 species; *kalatl* (frogs and toads), including 10 ethnocategories representing 29 species; and *ayotsi* (turtles), including one ethnocategory representing two species. Serpents are the most representative group; these were frequently mentioned in free lists and have the highest number of subcategories of classification (21) compared with lizards, turtles, and amphibians. Through image stimuli (photographs), we identified subcategories not identified during the interviews.

### *Kohuatl* (serpents)

#### Ethnocategories and cultural relevance

**Fig. 2:**
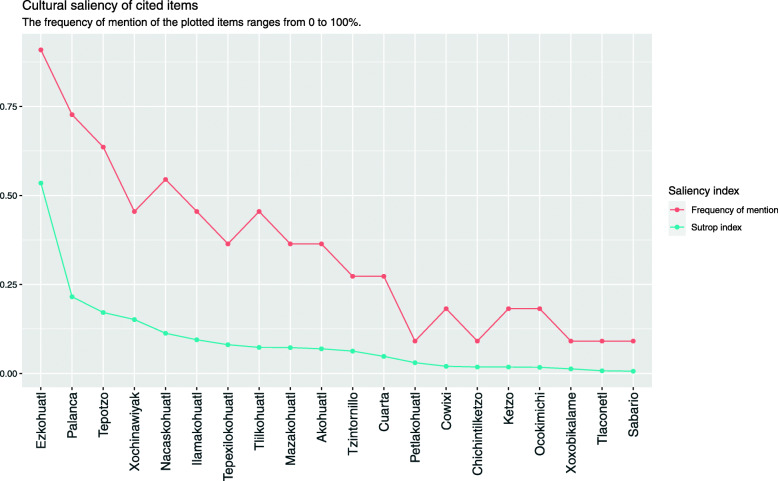
Frequency and order of mention, indicating that snakes are the most culturally relevant group for adult people

Adult people mentioned 18 subcategories, 13 of them mentioned in the free lists. Based on these data, we calculated the index of cultural relevance, serpents being the most important group, outstandingly the group *ezkohuatl* (0.5348) (including 10 spp.), *palanca* (0.2155) (including two species), *tepotzo* (0.1713) (including six species), *xochinawiyak* (0.1515) (including one species), and *nakaskohuatl* (0.1129) (including two species) (Fig. 2; Table 1).

**Table 1:** Number of snake species included by each social group in each of the ethnocategories. Adults 27 to 74 years old, young people (12 to 15 years old) and children (6 and 12 years old)

Náhuatl name		Spanish name	Total spp included		
			Adults	Young people	Children
1.	_______	Cinta/cuarta	7	^a^	
2.	***Akohuatl***	_______	5	1	
3.	***Cajfenkohuatl***	_______		1	
4.	***Kochipi***	Dormilona	2		
5.	***Kohuatl***	Serpiente	9		
6.	***Ezkohuatl***	Coralillo	10	2	3
7.	***Ilamakohuatl***	_______	6	1	4
8.	***Mazakohuatl***	_______	1		2
9.	***Nakaskohuatl***	Orejona	2	1	
10.	***Naranjaskohuatl***	_______		1	
11.	***Palanca***	_______	2		1
12.	***Palane***	Cinta venenosa	1		
13.	***Petlakohuatl***	_______	1		
14.	***Petlasolkohuatl***	_______	1		
15.	***Quimichkohuatl***	_______	1	1	
16.	***Tenexkohuatl***	_______	1		1
17.	***Tepexilokohuatl***	_______		1	3
18.	***Tepotzo***	_______	6	1	2
19.	***Tepotzonsi/Tsintornillo***	Sin tornillo	2	1	
20.	***Tlilkohuatl***	Ratonera	2	1	1
21.	***Xochinawiyak***	_______	1	1	

^a^ The ethnocategories did not include any of the species shown in the photographs

**Fig. 3:**
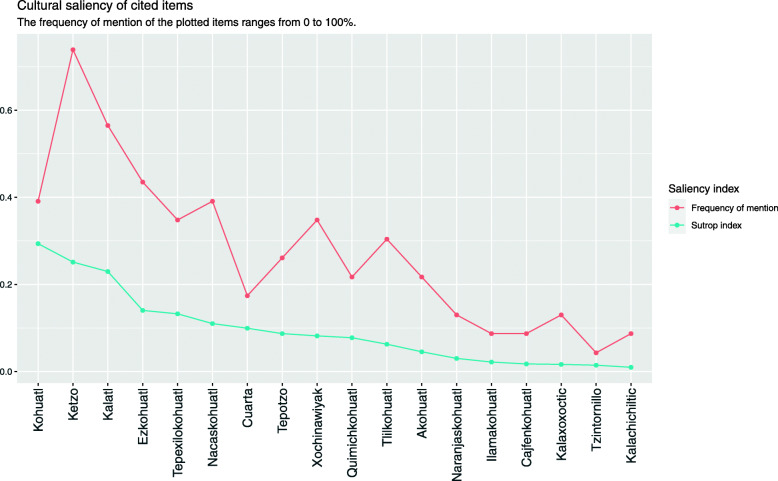
Frequency and order of mention, indicating that snakes are the most culturally relevant group for young people

Unlike adults, young people included in their free lists three main categories and some subcategories for classifying herpetofauna. Nevertheless, serpents are also the most notable. They listed 13 ethnocategories, the most culturally relevant being *ezkohuatl* (0.1403) (two species), *tepexilokohuatl* (0.1325) (one species), and *nakaskohuatl* (0.11) (one species) (Fig. 3).

Children recognized nine subcategories of serpents (Table 1), *ilamakohuatl* (four species), *ezkohuatl* (three species), and *tepexilokohuatl* (three species), having the highest cultural relevance.

Serpents with the greatest cultural relevance are, in general, those considered more poisonous. Biological, morphological, and ecological knowledge on serpents has created communitarian knowledge that allows recognizing and protecting from them. *Ezkohuatl*, *palanca*, *tepotzo*, *xochinawiyak,* and *nakaskohuatl* are identified through characteristics associated to potentially dangerous serpents, for instance, dark colours, figures in the back, robustness, and large size are the main features of the poisonous serpents’ "family", which are commonly eliminated when found. There is an exception with the *tlilkohuatl*, a large, black serpent, about which we will comment ahead.

#### Material and non-material importance (use, management, perceptions, beliefs, and legends)

Use of serpents is mainly medicinal; this was mentioned by all people interviewed and in workshops with children and young people. For this use, people collect the serpents alive and put them in jars that are then filled with sugarcane aguardiente. This aguardiente is drunk for treating serpent and other poisonous animal bites, toothache, skin infections, and intoxications. In cases of intoxications, people should drink one or several small glasses. Cases of snakebite require variable and more complex procedures: in some cases, people practice a tourniquet in the affected zone, make cuts to extract poison and take one or several times of the prepared aguardiente. For treating toothache, a piece of cotton is wetted in aguardiente, then pressing on the affected tooth with it (several persons interviewed said that this treatment may weaken and break the affected tooth). For insect bites and skin infections, the prepared aguardiente is topically applied on the affected part. Although the forms of using this medicine are widely known, there are different criteria in relation to doses and regimes of administration.

**Fig. 4:**
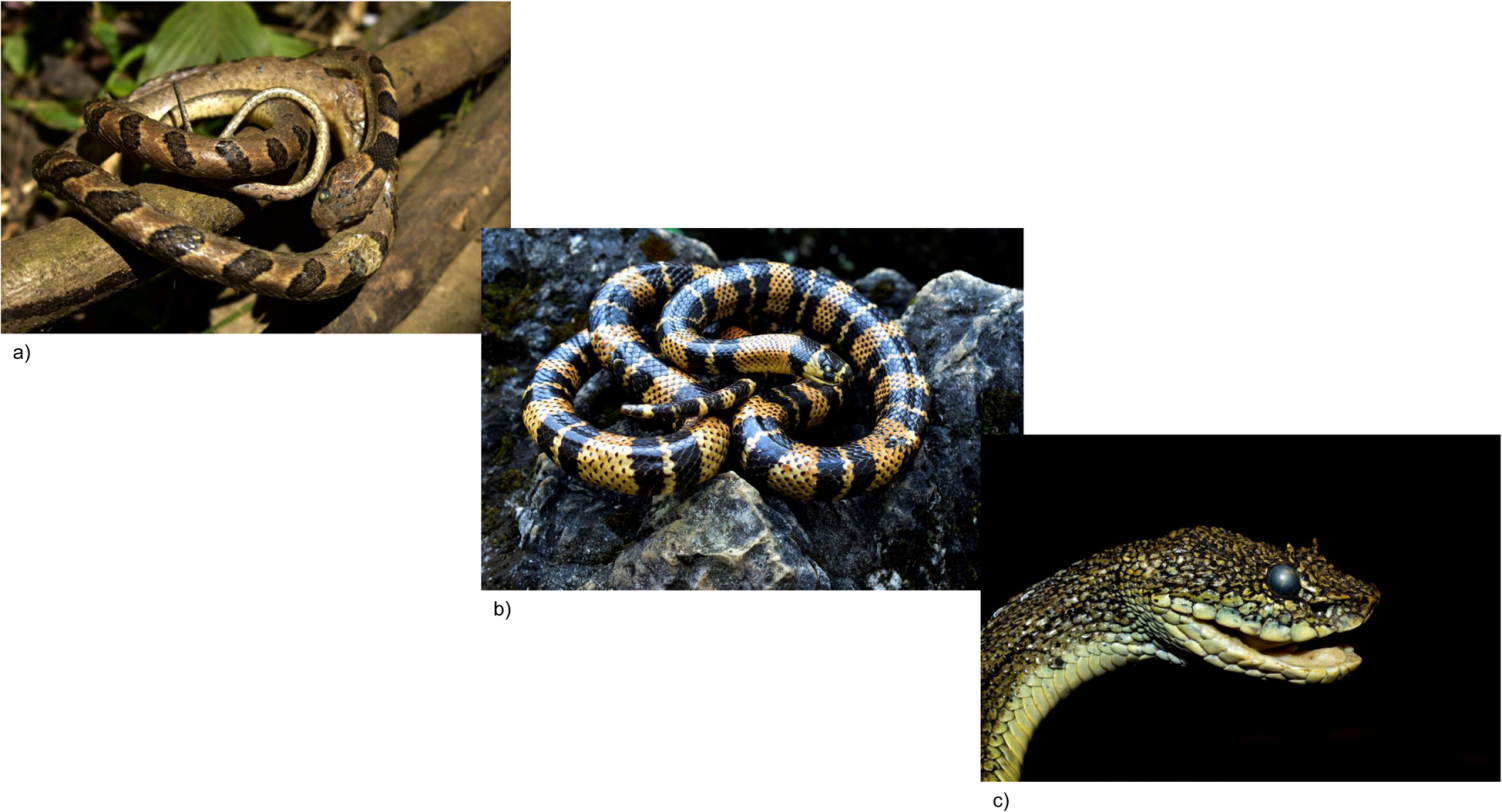
Snake species most represented in medicinal use: **a** *Leptodeira polisticta*, **b** *Lampropeltis polyzona*, **c** *Opryacus undulatus*

Eleven species of medicinal use were identified, classified in 5 ethnocategories (Table 2). Two species are endemic and endangered, the *nakaskohuatl* (*Ophryacus undulatus*) considered as vulnerable by International Union for conservation of Nature (IUCN), and *petlasolkohuatl* (*Thamnophis sumichrasty*) in the category of threatened by the NOM-059-SEMARNAT-2010. The most represented species were *palane* or cinta venenosa (*Leptodeyra polysticta*) with four specimens, *ezkohuatl* or coralillo (*Lampropeltis polizona*), and *nakaskohuatl* with three specimens each (Fig. 4).

**Table 2:** Species of snakes determined and the ethnocategories to which they correspond, numbers of specimens reviewed, and their conservation status.

Snake species		Nahuatl name	Spanish name	No. individuals	Status NOM-059-SEMARNAT-2010^**a**^	IUCN^**b**^	Endemism
1	*Pliocercus elapoides*	***Ezkohuatl***	Coralillo	1		Lc	
2	*Leptodeira polysticta*	***Palane***	Cinta venenosa	4		Lc	
3	*Lampropeltis polyzona*	***Ezkohuatl***	Coralillo	3		Lc	
4	*Imantodes cenchoa*	______	______	2	Pr	Lc	
5	*Ophryacus undulatus*	***Nakaskohuatl***		3	Pr	Vu	En
6	*Micrurus elegans*	***Ezkohuatl***	Coralillo	1	Pr	Lc	
7	*Micrurus nigrocinctus*	***Ezkohuatl***	Coralillo	1		Lc	
8	*Tropidodipsas sartori*	***Ezkohuatl***	coralillo	1		Lc	
9	*Thamnophis sumichrasti*	***Petlasolkohuatl***		1	A	Lc	En
10	*Spilotes pullatus*	***Xochinawiyak***	___	1		Lc	

^a^ *Pr*: subject to special protection; *A*: threatened

^b^ *Lc* least concern, *Vu* vulnerable

**Fig. 5:**
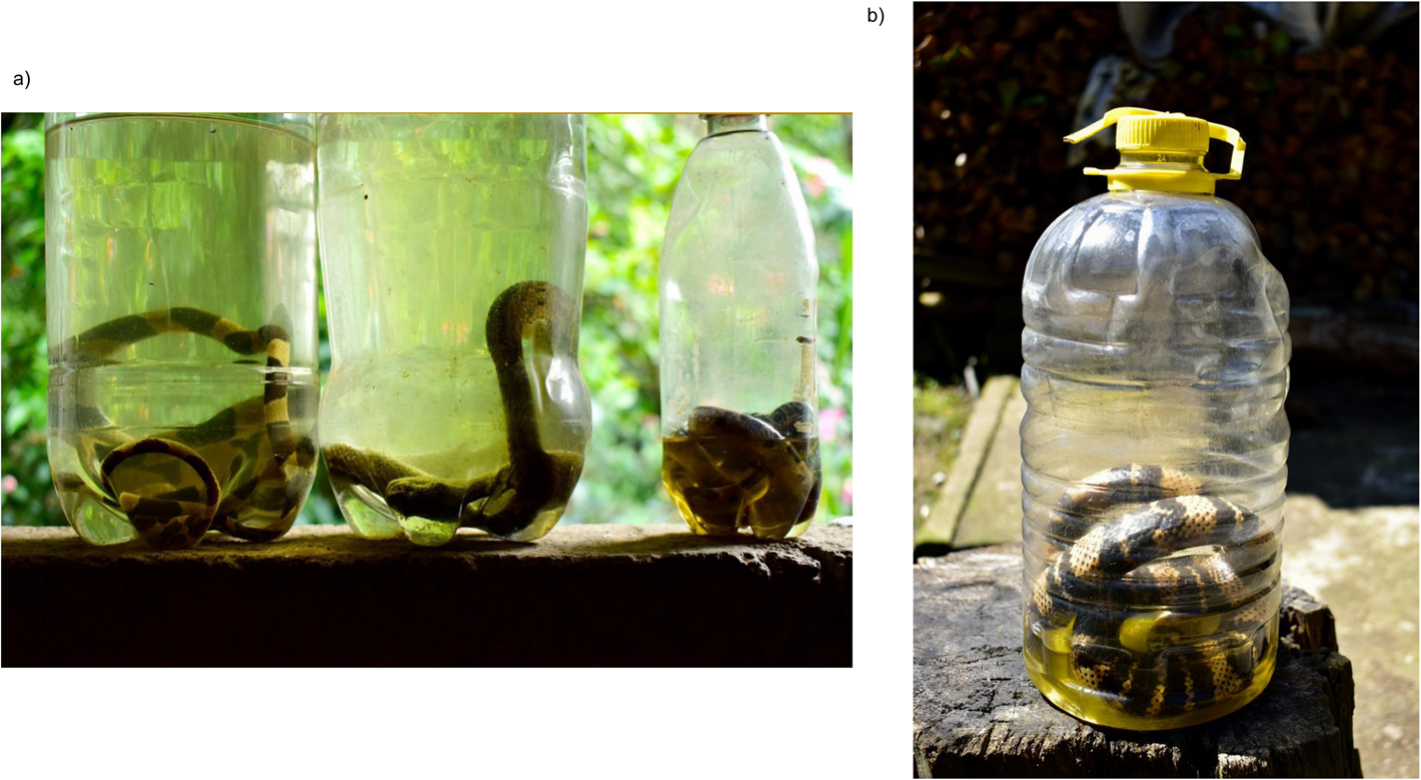
Medical use: **a** *Imantodes cenchoa*, *Opryacus undulates*, and *Leptodeira polysticta;* **b** *Lampropeltis polyzona*

Serpent medicines are commercialized (occasionally donated), and sometimes the serpents are sold alive (Fig. 5), but the latter is rare since the snake bites in the study area indicate that capturing a serpent is a risk more than an economic benefit. However, we documented that some persons maintain *ezkohuatl* and *palane* in captivity to sell them. One interviewee said to have learned this activity from another person in the community, but this is rather an opportunistic activity. It is carried out with a stick including a pitchfork in the tip and a bottle where the serpent is deposited. We recorded the ornamental use of serpent skin, but this was rare (6.25%).

People referred to several recent snake bite accidents that happened in the zone; while men were working in the field, snakebites were in arms and hands, and the consequence was the loss of moving capacity of hand and/or fingers. All people interviewed said to have killed serpents, and children and young people in workshops referred to have done it as well. Elimination of serpents is considered an action in favour of security for men in their labour and protection for children, women, and domestic animals. Different techniques with different degrees of complexity of serpent elimination were reported; these ranged from killing them with a machete or stones, to burning them (to scare away other snakes) or hanging them on a stick (for the birds to eat); parents teach their children how to do it. Collection of alive animals and their maintenance in captivity are also practiced as mentioned before, but these practices are rare compared with elimination. Tolerance is practiced with some species, mainly the *tlilkohuatl* (*Drymarchon melanurus*), nearly 50% of people interviewed consider that this serpent is inoffensive and benefit their areas of activity since they are good regulators of populations of other mammals like mice, and gophers, and even other poisonous serpents (see next quote). One interviewee said to have practiced relocation of *tlilkohuatl* specimens to their maize field to protect it against rodents.

“The *tlilcuhuatl* is very clever to kill other snakes, even when these are big ones” (Rutilio 74 years old)*.*

In relation to non-material importance of serpents, we recorded that according to old people, some serpents have moral attributes; for instance, the *tlilkohuatl*, *akohuatl*, *cinta,* and *ezkohuatl* have the capacity to reprimand people when they work angry, or they are lazy, or speak bad words (serpents may appear in the paths hitting people like a whip or rolling up on their feet thus causing fright).

“It is black, I think pure black (the *tlilkohuatl*). And people say that if you get angry and you go somewhere, that snake goes after you to roll up your feet. That happened to my mum.” (Sonia 32 years old).

Elimination of serpents may have consequences; for instance, according to people, killing a *mazakohuatl* may cause heavy rains. According to old people, Quetzalcoatl lives in the mountains surrounding the villages; where he is present, water is never lacking in 5 to 10 years. Quetzalcoatl comes and provides abundant harvest in maize fields, coffee plantations, flowers, and fruit in the forest.

“They (grandparents) realize that… well, they say that when Quetzalcóatl appears, the good time will be present for five to ten years. Then, it moves to another place where it will provide benefit.” (Damián 60 years old)

These beliefs are being lost among children, young and even adult people. Instead, negative beliefs are gaining terrain; for instance it is common that people think that serpents are aggressive, throw poison, cause bad dreams or that only by touching them is enough to cause damage in the skin. Negative beliefs and snake bite accidents have increased the general perception of serpents as dangerous animals that should be eliminated.

### *Ketzo* (lizards and salamanders), *kalatl* (frogs and toads), and *ayotsi* (turtles)

#### Ethnocategories and cultural relevance

Although lizards, turtles, and amphibians are less relevant than serpents in the Nahua community, people interviewed recognize several species living in the communities studied or in neighbouring areas. Their identification is based on morphology (mainly colours, forms, and sizes), ethology (behaviour, sounds produced, and habits) and ecology (distribution, habitat types, seasonality, and food habits).

For the *ketzo* category (lizards and salamanders), adult people recognize 10 subcategories (Table 3), but only *cowixi* (0.0202) (six species), *ketzo* (0.0182) (six species), *ocokimichi* (0.0173), *tlalconetl* (0.0076) (seven species), and *sabario* (0.0065) (two species) were mentioned in the free lists with low values of cultural relevance (Fig. 2). Young people mentioned 4 ethnocategories, but *ketzo* (0.2513) was the only one that appeared in the free lists with a high index of cultural importance (Fig. 3), recognizing three species. Children recognized three subcategories, with *ketzo* (three species) and *tlalconetl* (three species) including more species. The ethnocategories *tlalconetl*, *tetlina*, *topitzi*, and *tlalkuitla* include both salamanders and lizards.

**Table 3:** Number of species of lizards, amphibians, and turtles included by each social group in each ethnocategory

Náhuatl name		Spanish name	Total of spp included		
			Adults	Young people	Children
**Lizard/salamander**					
1.	***Tlaconetl***	______	7	2	3
2.	***Ketzo***	Lagartija (lizard)	6	3	3
3.	***Cowixi***	______	6		
4.	***Tlalkuitla***	______	4		
5.	***Topitzi***	______	4		1
6.	***Tetlina***	______	3		
7.	______	Sabario	2		
8.	***Inantetl***	______	1		
9.	***Ocokimichi***	Lagartija	^a^	^a^	
10.	***Chichintilketzo***	Lagartija gris (grey lizard)	^a^		
11.	***Mikakimichi***			^a^	
**Frog/toad**					
12.	***Kalatl***	Rana (frog)	24	9	1
13.	***Kalatlpipitzo***			3	
14.	***Kalaxoxoctic***	Rana verde (green frog)		1	
15.	***Kalachichiltic***	Rana roja (red frog)		1	
16.	***Kalame***			3	1
17.	***Kalatera***			1	
18.	***Kalapalanki***			2	1
19.	***Zibatl***	Rana	^a^		
20.	***Xoxobikalame***	Rana azul (blue frog)	^a^		
21.	***Okichtli***	Sapo	^a^		
**Turtle**					
22.	***Ayotsi***	Tortuga (turtle)	2	2	1

^a^ The ethnocategories did not include any of the species shown in the photographs

The categories *cowixi* and *sabario* consider classificatory criteria associated to the habitat of animals. People recognize two types of *cowixi* and two of *sabario* (the ones from the ground and the ones living in trees). Two persons said that *cowixis* and *sabarios* originate from the different poisonous serpents. Some lizards are considered poisonous, and people transmit information and practices to recognize them in order to protect people.

In the category *kalatl* (frogs and toads), adult people recognize four subcategories, but only *xoxobikalame* (0.013) appeared in the free lists with low value in the index of cultural relevance (Fig. 2). *Kalatl* was the only category in which people included species (24 spp.) (Supplementary material 1). Young people mentioned seven subcategories (free lists and images). In free lists, *kalatl* (0.2296) has a high index of cultural relevance (Fig. 3), and it is the ethnocategory with more species (nine), while *kalaxoxoctic* (0.0163) and *kalachichiltic* (0.0097) had low relevance. Children indicated three categories of frogs and toads, with one species each. The category *ayotsi* was less relevant, but people recognized two species.

#### Material and non-material importance (use, management, perceptions, beliefs, legends)

Five and six persons (nearly 31.2% and 25% of people interviewed) indicated that vocalization frogs or other, *tetlina*, *topitzi*, and *tlalkuitla* sounds, respectively, are indicator of the rainy season arrival.

“Yes, it (the *tetlina*) warns that something is going to happen with rain. This, the *tetlina*, warns, that is why it is always in a cool place.” (Celestino 65 years old).

**Fig. 6:**
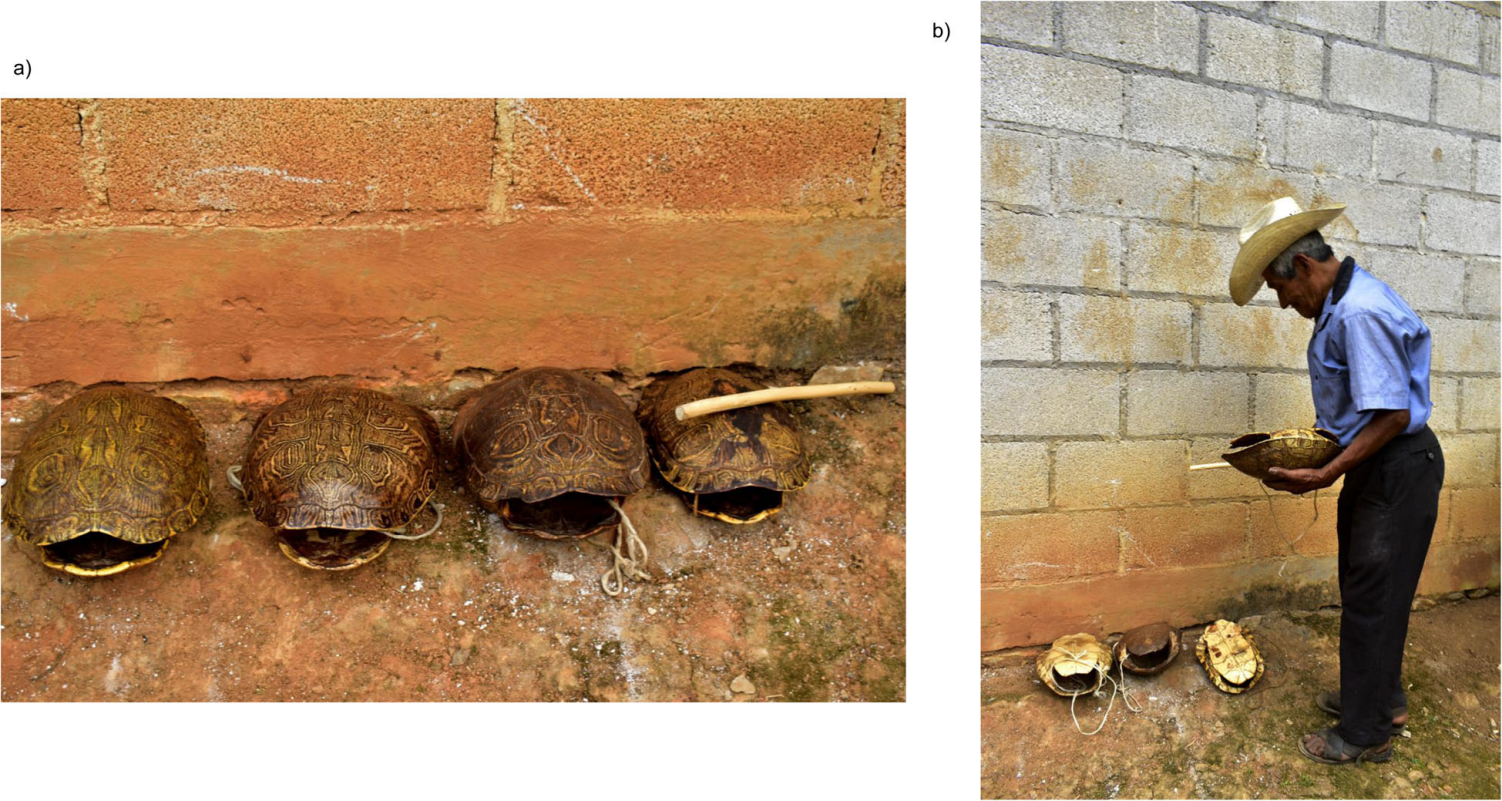
Instrumental use of the turtle (*Trachemys sp)*

Six persons (about 37.5% of people interviewed) said that shells of turtles (*Trachemys* sp.) are used as musical instruments for the holy Thursday feast during the Way of the Cross, but their use is decreasing (Fig. 6). One of the interviewees said that his grandfather maintained a lizard as a pet for several years (we could not identify the species).

Eight persons (nearly 50% of people interviewed) perceive that *ocokimichi* and *cowixis* are poisonous animals. They said that some frogs cause skin irritation and rush.

In relation to management, we found that in general, lizards, salamanders, and frogs are tolerated, even the lizards considered poisonous, the latter are not eliminated but only driven away. Five persons (nearly 31.2% of people interviewed) referred to different beliefs associated to *tlaconetl*, *topitzi*, and *ketzos*. They think that *tlaconetl* and *topitzi* could get women pregnant, even drunk people sleeping in the field, but if these animals are eliminated, a relative may die. In relation to *ketzos*, people said that these animals may suck blood, but if these are eliminated, the action could cause an epileptic attack. One person interviewed said that a frog (we could not identify the species based on the description) has the capacity of foreshadow longevity of people. When the frog is found (which, according to the interviewed is unusual), it is put underground, then after the labour, when the person returns, this one unearths the frog; if it is alive, it is a signal that the person will have long life.

## Discussion

**Fig. 7:**
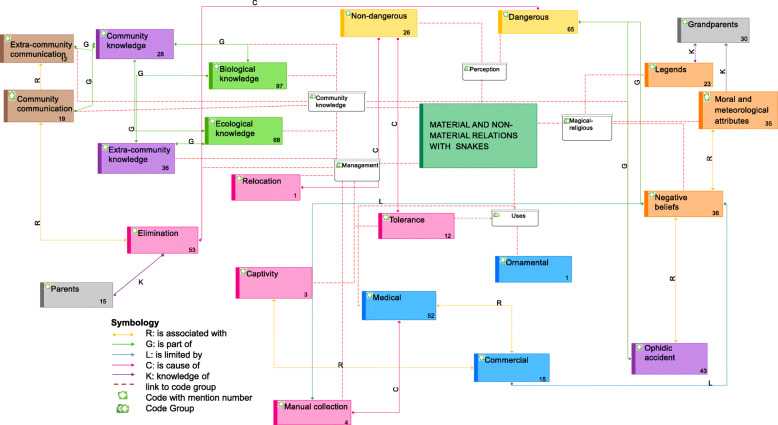
Material and non-material relationships between the Nahua people and snakes (green). The community knowledge that sustains these relationships has been created by several factors: biological (including morphological and ethological) and ecological knowledge (green), intra and extra-community knowledge (purple), and intra and extra-community communication (brown). Material relationships are shown in the different forms of management (pink) and use (blue), while non-material relationships are shown in magical–religious aspects (orange) and perceptions (yellow). Social group that has transmitted most of this information (grey). The lines show the relationships that exist between the different aspects

**Fig. 8:**
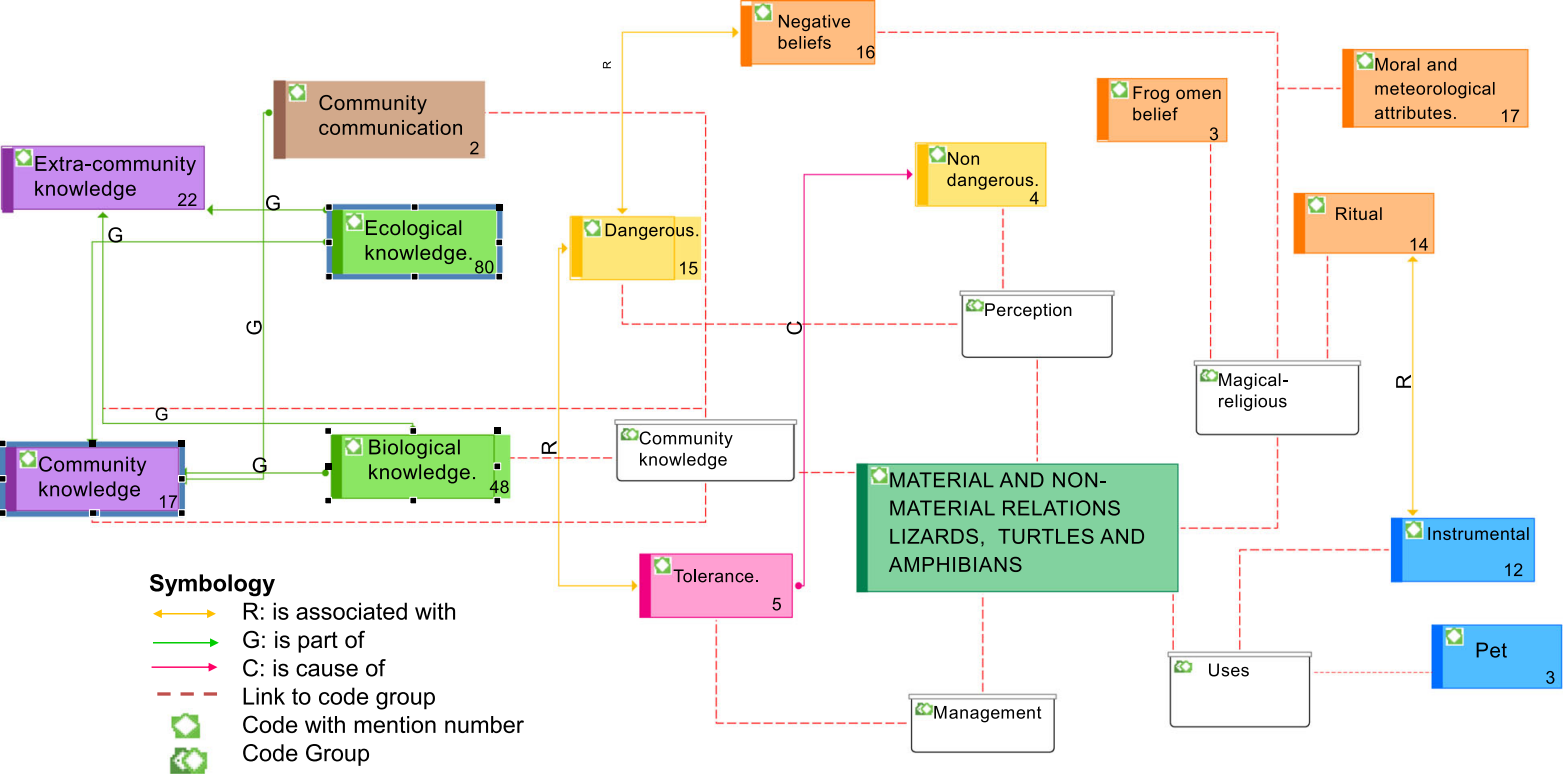
Material and non-material relationships between Nahua people and lizards, turtles, and amphibians (green). The community knowledge that sustains these relationships has been created by various factors: biological (including morphological and ethological) and ecological knowledge (green), intra and extracommunity knowledge (purple), community communication (brown). Material relationships are shown in the different forms of management (pink) and use (blue), while non-material relationships are shown in magical–religious aspects (orange) and perceptions (yellow). The lines show the relationships that exist between the different aspects

The relations between the Nahua people from Aticpac and Xaltepec and the local herpetofauna combine ancient and new elements in the material and non-material contexts (Figs. 7 and 8). For understanding these relations, the idea of Nahua contemporary knowledge [14] is helpful, since it reflects the knowledge dynamism and helps to recognize and understand the integrity of diverse sources of coexisting knowledge [14, 17] as well as the importance of this knowledge in the present-day world. We identified that although knowledge, beliefs, and practices have a root in the Nahua local ecological knowledge, these are influenced by new elements from direct observation, learning from other people, communitarian intra and inter communication and the media (internet, TV), books, and classes in the school, as well as customs, beliefs, and knowledge from people from outside the community. People from the communities that have studied have periodic interaction with other people from several communities of the region in the regional markets. In addition, some persons have migrated or have relatives living out of the region. All these factors have had influence on the construction, transformation, renovation, and transmission of communitarian knowledge and have been determinant in the contemporary perception and practices on the herpetofauna of the zone as shown in the following quote.

“It is what I call Quetzalcóatl, and that is why I realized it is true, because I even remember when I was a child and read books of literature and history, where it was explained that ancient people adored it very much.” (Damián 60 years old).

### Ethnocategories

In Aticpac and Xaltepec, we recorded four main categories of Herpetofauna: *kohuatl*, *kalatl*, *ayotzi*, and *ketzo* with 21, 10, one, and 11 ethnocategories, respectively. The list reported here is a first approach to an ethnoherpetological list of the zone since participatory sampling is still required. However, our results provide relevant ethnoherpetological data; for instance, three general categories (serpents, frogs and toads, and turtles) are similar to the Linnean classification, but the category *ketzo* includes lizards and salamanders. This is similar in other regions; for example, in Sierra Norte of Puebla, the Nahua have one ethnocategory—*okuiltsin* (“bugs”)—which not only includes small lizards and salamanders, but also include several invertebrates [37]. Therefore, the ethnocategory present in Aticpac and Xaltepec tends to be more specific.

Identification and classification of herpetofauna by the Nahua people are based on morphological, ethological, and ecological aspects, which reflects the deep knowledge that local people have on animals studied. It is also relevant that there is a specific category for frogs and toads, since previous studies in the region with the Cuicatec and the Nahua from the Sierra Norte of Puebla reported that all frogs, salamanders, and small lizards are classified together in a category [24, 37].

### Material importance: uses, management, and perception

The subcategories *kohuatl* (serpents), followed by *ketzo* (lizards and salamanders), were the most represented in both material and non-material aspects. It has been documented that at international [52] and national [30] levels, reptiles have higher utilitarian importance than amphibians. For Mexico, it has been reported that 11% of reptiles and 8% of amphibians are used mainly for medicine and food [30]. The prevalence of these uses were reported previously in the Tehuacán-Cuicatlán Valley [23]. The latter study reports that the Nahua of the region make use of herpetofauna mainly for medicinal purposes [23], this information is substantially complemented with the data documented in our study. Not only in relation to the types of illnesses attended, but also the list of species of serpents used for that purpose.

Although remedies based on serpents include different illnesses, the most common is treating snake bite. We recorded 10 species of serpents used for remedies; among them are colubrid, viperid, and elapid. According to the Norma Oficial Mexicana and the IUCN, two of these species are endangered: the colubrid *Thamnophis sumichrasty* is threatened, and the viperid *Ophryacus undulatus* is vulnerable. The variation in the techniques of attending snake bite accidents include practices that could generate complications, while variation in species used in ethnomedicine include species without medicinal effectiveness (colubrid), all of which represent problems for biocultural conservation and public health that should be attended. Since 2017, the World Health Organization recognized that problems associated to serpent bites are among the main unattended tropical illnesses [53].

In relation to other uses of herpetofauna, such as food, we found that differently to information reported from the Nahua in the state of Morelos and the Cuicatec in the Tehuacán Valley [24, 38], people of Aticpac and Xaltepec do not consume any reptile; and associated to subsistence hunting, before cooking an animal, they check to be sure that the animal hunted has not eaten a serpent recently. Although in rare practices, we recorded the commercial use of serpents or remedies prepared with them, including the ornamental use of their skin, the use of a lizard as a pet, and the use of turtle shells as musical instruments. All this information complements the previous reports [23].

Management of herpetofauna by the Nahua is variable and closely related to perceptions and beliefs. Previous studies reported the collection of alive serpents in the Tehuacán-Cuicatlán Valley [23]. We in addition documented other practices, like the maintenance in captivity of serpents *(ezkohuatl* and *palane*) and the elimination as a prevalent form of management during casual encounters. Cubides and Alarcón [54] mention that in general, the elimination of serpents is motivated by fright rather than a feeling of antipathy. Our study supports this idea, and, on the contrary, we documented other forms of interaction like tolerance or relocation of the *tlilkohuatl* and the tolerance of lizards and salamanders even when some of them are considered poisonous, but less than serpents.

### Non-material importance: meteorological value, moral, and legends

Several Mesoamerican cultures consider frogs and toads having meteorological value to announce the proximity of the rainy season [25]. In the Tehuacán-Cuicatlán Valley, previous studies recorded that the Nahua people of the region confers this value also to some birds and mammals [23]. We add to these information frogs, toads, and some salamanders and lizards (the *tetlina*, *topitzi*, and *tlalkuitla*).

Toledo and Barrera-Bassols [7] used the term biocultural memory to refer to the knowledge transmitted from generation to generation, a process that establish relations of coexistence. The “... *final product of this process is currently present in minds and hands of men and women that conform…the indigenous peoples”* [7]*.* During this research, we found that the moral value of transmitting knowledge is mainly an old people attribute. Elders say that serpents have the faculty of reprimanding bad behaviour people, and this is similar to what has been reported in other regions like among the Nahuas of Sierra Norte de Puebla and the Lacandon in Chiapas [37, 55]. Old people are the ones remembering that Quetzalcoatl, a pre-Columbian deity, is around and provides good harvests. In some cases, the moral or meteorological value is indirect. For instance, the *tlalconetl* and *topitzi* get drunk people pregnant or heavy rains are caused by killing *mazakohuatl*. These aspects of the Nahua worldview regulate people’s behaviour and promotes respect and coexistence with other species. Unfortunately, these forms of conceiving and relating with herpetofauna are progressively less frequent (see Table 1) and suggests aspects to attend in an agenda for biocultural conservation.

### Biocultural conservation

The *in situ* biological conservation involves several approaches (genetic variation, species diversity, ecosystem maintenance). The *in situ* biocultural conservation is probably the proposal that requires the highest integration since it involves actions needed for biological and cultural conservation in social, political, and economic contexts [56]. *In situ* biocultural conservation recognizes the leading role of peasant and indigenous communities to manage and conserve biodiversity since they are the main stewards of biodiversity of the world [1, 2, 6, 7]. A main task in this direction is reinforcing the ethnic identity and strategies to stop losing indigenous knowledge systems and recovering them wherever possible as well as enhancing recognition, understanding and respect of pluriculturality [57]. Due to the loss of knowledge systems, beliefs, and practices, the form of interrelation between people and herpetofauna changes, as illustrated in this study. Some aspects derived from this study that deserve attention are for instance the negative beliefs, the generalized elimination of serpents, and adequate attention to snake bites.

For attending the high amount of negative beliefs associated to herpetofauna, it is indispensable to counterbalance the myths considering reptiles and amphibians as animals that seek to damage people. Socializing current scientific knowledge along with local knowledge, beliefs, legends, and testimonies of old people would be crucial. This would allow restructuring perception on these animals in new generations, at the same time make possible the reinforcing of ethnic identity, local values, and knowledge of herpetofauna that have been present for centuries and that have been discriminated, devaluated, and negated [58]. A dialogue of knowledges, horizontal among worldviews, contexts, and different biocultural realities, would be favourable to interchange knowledge and views, collective reflexion, re-contextualization, and re-signification of knowledge [59, 60]. Socializing knowledge that already exists is important as well as complementing it with other scientific information about the importance of herpetofauna in ecosystems, and the consequences of its loss, the way of identifying the different serpent species, their importance in ethnomedicine, and the methods of modern medicine for treating serpent bites are all needed. However, maintenance of biodiversity is deeply linked to maintain cultural processes that make use of it; therefore, promoting its valuing and use is crucial for *in situ* biocultural conservation [4]. We recognize that medical knowledge and practices based on herpetofauna have been present since centuries ago and have been helpful to face health problems [23, 52, 61]. In fact, serpent poisons are now part of the modern medicine for treating cancer, epilepsy, poliomyelitis, rheumatism, arthritis, and other illnesses [52]. Therefore, the knowledge and practices documented in this study may have extraordinary value. The World Health Organization has pointed that serpent bites are a priority problem to attend in the tropics, and local knowledge may make important contributions [62].

## Conclusions

In the communities of Aticpac and Xaltepec, there is an important body of Nahua knowledge on herpetofauna of the region studied, which has roots in local knowledge and has been dynamic, influenced by new internal knowledge and experiences, as well as external information, what we call contemporary. This knowledge influences significantly material and non-material relations between people and herpetofauna. Serpents are the group with higher cultural relevance, with a marked negative perception towards them because of fright, determining that the main form of interaction is their elimination, but for most, herpetofauna tolerance is the dominant relation. Medicinal use of serpents is the most important, but we recorded other uses of the local herpetofauna. Perceptions, beliefs, and legends influence the relation of people with these animals. In old people and some young adults, some elements of the Nahua biocultural memory are present, providing information and values to the whole community, which, together with scientific information, would contribute to design strategies of *in situ* biocultural conservation.

## Supplementary Information

**Additional file 1: Supplementary Material 1.** Ethnoherpetological Listing.

## Data Availability

The datasets used and/or analysed during the current study are available from the corresponding author upon reasonable request.

## Abbreviations

ECOSUR: El Colegio de la Frontera Sur
IIES: Instituto de Investigaciones en Ecosistemas y Sustentabilidad
TEK: Traditional ecological knowledge
TCV: Tehuacán-Cuicatlán Valley
NOM: Mexican Official Norm
IUCN: International Union for conservation of Nature
