# Supplementary material for: Nahua biocultural richness: an ethnoherpetological perspective
Source: J Ethnobiol Ethnomed. 2021 May 12;17:33. doi: 10.1186/s13002-021-00460-1 (PMC8115869; doi:10.1186/s13002-021-00460-1)
Supplement: Supplementary file 1 — Additional file 1: Supplementary Material 1. Ethnoherpetological Listing. [file 13002_2021_460_MOESM1_ESM.docx]

Supplementary Material 1. Ethnoherpetological Listing

| **Náhuatl** | **Spanish** | **Total *spp.* included** | **Spp. codes** | **Scientific name** |
| --- | --- | --- | --- | --- |
| **Kohuatl (snakes)** | | | | |
| **_______** | Cinta/cuarta | 7 | 99, 107, 113, 114, 127, 136 y 138 | *Masticophis mentovarius* (Duméril, Bribon and Duméril, 1854)*, Enulius flavitorques* (Cope, 1869)*, Imantodes cenchoa* (Linnaeus, 1758)*, I. gemmistratus* (Cope, 1860)*, Rhadinea hesperia* (Bailey, 1940)*, Tantilla bocourti* (Günther, 1895) y *T. rubra* (Cope, 1876)*.* |
| **________** | Palanca | 2 | 151 y w/c3 | *Agkistrodon bilineatus* (Günther, 1863) y *Bothrops asper* (Garman 1884) |
| **Akohuatl** | _______ | 6 | 99, 102, 108, 113, 118 y 126 | *Masticophis mentovarius* (Duméril, Bribon and Duméril, 1854)*, Conopsis acuta* (Cope, 1886), *Ficimia olivácea* (Gray, 1849)*, Imantodes cenchoa* (Linnaeus, 1758)*, Leptophis diplotropis* (Günther, 1872) y *Pseudoleptodeira latifasciata* (Günther, 1894) |
| **Cajfenkohuatl** | ______ | 1 | 127 | *Rhadinea hesperia* (Bailey, 1940) |
| **Kochipi** | Dormilona | 2 | 99 y 106 | *Masticophis mentovarius* (Duméril, Bribon and Duméril, 1854) y *Drymarchon melanurus* (Duméril, Bribon and Duméril, 1854) |
| **Kohuatl** | Serpiente | 9 | 104,108,119,139,144,151,124,150 y 123 | *Conophis lineata (*Kennicott, 1859*), Ficimia olivácea* (Gray, 1849)*, Leptophis mexicanus* (Duméril, Bribon and Duméril, 1854)*, Thamnophis chrysocephalus* (Cope, 1885)*, Trimorphodon tau* (Duméril, Bribon and Duméril, 1854)*, Agkistrodon bilineatus* (Günther, 1863)*, Pseudoficimia frontalis* (Cope, 1864)*, Rena máxima* (Loveridge, 1932) *y Pituophis* deppei (Duméril, 1853) |
| **Ezkohuatl** | Coralillo | 11 | 100, 111, 115, 120,122, 134, 145, 146 147, 148 y 149 | *Tantilla melanocephala* (Peters, 1869)*, Geophis semidoliatus* (Duméril, Bribon and Duméril, 1854)*, Lampropeltis polizona* (Lacépede, 1788), *Ninia sebae* (Duméril, Bribon and Duméril, 1854)*, Oxyrhophus petolarius* (Linnaeus, 1758)*, Sonora michoacanensis* (Duges, 1884)*, Tropidodipsas sartorii* (Cope,1863)*, Micrurus diastema* (Duméril, Bribon and Duméril, 1854), *M. elegans* (Jan, 1858)*, M. laticollaris (*Peters, 1869) y *M. pachecogili* (Campbell, 2000)*.* |
| **Ilamakohuatl** | _______ | 8 | 106, 112, 136, 152, 154 ,155, 156, w/c 1 | *Drymarchon melanurus* (Duméril, Bribon and Duméril, 1854)*, Hypsiglena torquata* (Günther, 1860)*, Tantilla bocourti* (Günther, 1895)*, Crotalus culminatus* (Klauber,1952)*, C. molossus* (Baird y Girard, 1853)*, C. ravus* (Cope, 1865)*, C. scutulatus* (Kennicott, 1861)*,* y *Metlapilcoatlus nummifer* (Rüpper,1845) |
| **Mazakohuatl** | _______ | 1 | 98 | *Boa constrictor* (Linnaeus, 1758) |
| **Nakaskohuatl** | Orejona | 2 | 157 y 158 | *Mixcoatlus melanurus* (Müller, 1923) y *Ophryacus undulates* (Jan, 1859) |
| **Naranjaskohuatl** | ______ | 1 | 120 | *Ninia sebae* (Duméril, Bribon and Duméril, 1854) |
| **Palane** | Cinta venenosa | 1 | 116 | *Leptodeira polysticta* (Günther, 1895) |
| **Petlakohuatl** | _______ | 1 | 135 | *Spilotes pullatus* (Linnaeus, 1758) |
| **Petlasolkohuatl** |  | 1 | w/c2 | *Thamnophis sumichrasty* (Cope, 1885) |
| **Quimichkohuatl** | _______ | 1 | 106 | *Drymarchon melanurus,* (Duméril, Bribon and Duméril, 1854) |
| **Tenexkohuatl** | ______ | 2 | 99 y 143 | *Masticophis mentovarius* (Duméril, Bribon and Duméril, 1854)*,* y *Trimorphodon biscutatus* (Duméril, Bribon and Duméril, 1854) |
| **Tepexilokohuatl** | _______ | 3 | 118,119 y 124 | *Leptophis diplotropis* (Günther, 1872)*, L. mexicanus* (Duméril, Bribon and Duméril, 1854) y *Pseudoficimia frontalis* (Cope, 1864) |
| **Tepotzo** | _______ | 8 | 98, 103, 105,121, 132, 143 y 152,  w/c1 | *Boa constrictor* (Linnaeus, 1758)*, Conophis biserialis* (Taylor y Smith, 1942)*, C. nasus* (Günther, 1858)*, Oxybelis aeneus* (Wagler, 1824)*, Senticolis triaspis* (Cope, 1866)*, Trimorphodon biscutatus* (Duméril, Bribon and Duméril, 1854)*, Crotalus culminates* (Klauber, 1952) y *Metlapilcoatlus nummifer* (Rüpper,1845) |
| **Tepotzonsi/Tsintornillo** | Sin tornillo | 3 | 105, 109 y 143 | *Conophis nasus* (Günther, 1858), *Ficimia publia* (Cope, 1866) y *Trimorphodon biscutatus* (Duméril, Bribon and Duméril, 1854), |
| **Tlilkohuatl** | Ratonera | 3 | 99, 106 y 108 | *Masticophis mentovarius* (Duméril, Bribon and Duméril, 1854)*, Drymarchon melanurus* (Duméril, Bribon and Duméril, 1854) y *Ficimia olivácea* (Gray, 1849) |
| **Xochinawiyak** | Nauyaca | 1 | 135 | *Spilotes pullatus* (Linnaeus, 1758) |
|  |  |  |  |  |
| **Ketzo (lizards and salamanders)** | | | | |
|  |  |  |  |  |
| **______** | Sabario | 2 | 53 y 82 | *Basiliscus vittatus* (Wiegmann, 1828) y *Anolis quercorum* (Fitch, 1978) |
| **Chichintilketzo** | L. gris |  |  |  |
| **Cowixi** | ______ | 6 | 49, 53, 56, 78, 82 y 83 | *Abronia gramínea* (Cope, 1864)*, Basiliscus vittatus* (Wiegmann, 1828)*, Hemidactylus frenatus* (Schlegel, 1836)*, Anolis cymbops* (Cope, 1864)*, A. quercorum* (Fitch, 1978) y *A. sericeus* (Hallowell, 1856) |
| **Inantetl** | ______ | 1 | 41 | *Isthmura belli* (Gray, 1850) |
| **Ocokimichi** | ______ |  |  |  |
| **Ketzo** | Lagartija | 9 | 56, 66,69, 74,75, 78, 79, 80 y 93 | *Hemidactylus frenatus* (Schlegel, 1836)*, Sceloporus bicanthalis* (Smith, 1937)*, S. horridus* (Wiegmann, 1834)*, S. ochoterenae* (Smith, 1934) *, S. variabilis* (Wiegmann, 1834)*, Anolis cymbops* (Cope, 1864)*, A. microlepidotus* (Smith y Van Gelder, 1955)*, A. laeviventris* (Wiegmann, 1834) y *Aspidoscelis parvisocius* (Zweifel, 1960) |
| **Tetlina** | ______ | 3 | 41, 43 y 70 | *Isthmura belli (Gray, 1850), Pseudoeurycea leprosa* (Cope, 1869)*,* y *Sceloporus jalapae* (Günther, 1890) |
| **Tlalconetl** | Tlaconete | 8 | 41, 45, 47, 51, 85, 86, 87 y 88 | *Isthmura belli* (Gray, 1850)*, Thorius dubitus* (Taylor, 1941)*, T. schmidti* (Gehlbach, 1959)*, Celestus enneagrammus* (Cope, 1869)*, Marisora unimarginata* (Cope, 1862)*, Plestiodon brevirostris* (Günther, 1860)*, Scincella cherriae* (Cope, 1893) y *Scincella silvicola* (Taylor, 1937) |
| **Tlalkuitla** | ______ | 4 | 41,45, 47 y85 | *Isthmura belli* (Taylor, 1937)*, Thorius dubitus* (Taylor, 1941)*, T. schmidti* (Gehlbach, 1959) y *Marisora unimarginata* (Cope, 1862) |
| **Topitzi** | ______ | 5 | 40, 41, 45, 85 y 87 | *Ambystoma velasci* (Duges, 1888)*, Isthmura belli* (Taylor, 1937)*, Thorius dubitus* (Taylor, 1941)*, Marisora unimarginata* (Cope, 1862) y *Scincella cherriae* (Cope, 1893) |
| **Mikakimichi** |  |  |  |  |
| **Kalatl (frogs and toads)** | | | | |
| **Kalachichiltic** | Rana roja | 1 | 13 | *Craugastor rugulosus* (Cope, 1870) |
| **Kalame** | ______ | 3 | 7, 23 y 31 | *Craugastor alfredi* (Boulenger, 1898)*,* *Hyla eximia* (Baird, 1854) *y Smilisca cyanosticta* (Smith, 1953) |
| **Kalapalanki** |  | 2 | 21 y 39 | *Hyla arenicolor* (Cope, 1866) *y Lithobates zweifeli* (Frost y Webb, 1984) |
| **Kalatera** |  | 1 | 7 | *Craugastor alfredi* (Boulenger, 1898) |
| **Kalatl** | Rana | 26 | 17, 21, 22,25,32,3,4,5,6,8,9,20,26,27,28,31,33,7,10,  11,15,1,19,38,  23 y 18 | *Dendropsophus microcephalus* (Cope, 1866)*,* *Hyla arenicolor* (Cope, 1866)*, H. euphorbiacea* (Günther, 1859)*, Pachymedusa dacnicolor* (Cope, 1864)*, Tlalocohyla smithii* (Boulenger, 1901), *Incilius perplexus* (Taylor, 1943)*, I. valliceps* (Wiegmann, 1833)*, Rhinella marina* (Linnaeus, 1758)*, Hyalinobatrachium fleishmanni* (Boettger, 1893)*, Craugastor augusti* (Duges, 1879)*, C. galacticorhinus* (Canseco-Márquez and Smith, 2004)*, Exerodonta xera* (Mendelson and Campbell 1994)*, Sarcohyla* arborescandes (Taylor, 1939)*, S. bistincta* (Cope, 1877)*, Ptychohyla zophodes* (Campbell and Duellman, 2000)*, Smilisca cyanosticta* (Smith, 1953)*, Leptodactylus fragilis* (Cope, 1877)*, Craugastor alfredi* (Boulenger, 1898), *C. loki* (Shannon and Werler, 1955), *C. mexicanus* (Brocchi, 1877)*, Eleutherodactylus nitidus* (Peters, 1869)*, Incilius marmoreus (Wiegmann, 1833), Exerodonta smaragdina* (Taylor, 1940)*, Lithobates vaillanti* (Brocchi, 1977)*, Hyla eximia* (Baird, 1854) *y Rheohyla miotympanum* (Cope, 1863) |
| **Kalatlpipitzo** |  | 3 | 6, 16 y 23 | *Hyalinobatrachium fleishmanni (*Boettger, 1893)*, Agalychnis callidryas* (Cope, 1862) y *Hyla eximia* (Baird, 1854) |
| **Kalaxoxoctic** | Rana verde | 1 | 23 | *Hyla eximia* (Baird, 1854) |
| **Okichtli** | Sapo |  |  |  |
| **Xoxobikalame** | Rana azul |  |  |  |
| **Zibatl** | Rana |  |  |  |
| **Ayotzi (turtles)** | | | | |
| **Ayotzi** | **Turtles** | 2 | 159 y 160 | *Kinosternon integrum* (Le Conte, 1824) *y Trachemys* (Agassiz, 1857) *sp.* |

w/c: snakes without a code were not listed in the Sierra Madre del Sur herpetofauna, but their presence or possible presence in the field was recorded.
